# Profile of liver cholestatic biomarkers following prolonged ketamine administration in patients with COVID-19

**DOI:** 10.1186/s12871-023-02006-2

**Published:** 2023-02-07

**Authors:** Julie Henrie, Ludovic Gerard, Caroline Declerfayt, Adrienne Lejeune, Pamela Baldin, Arnaud Robert, Pierre-François Laterre, Philippe Hantson

**Affiliations:** 1 Department of Intensive Care, Cliniques St-Luc, Université Catholique de Louvain, Avenue Hippocrate, 10, 1200 Brussels, Belgium; 2 Department of Pathology, Cliniques St-Luc, Université Catholique de Louvain, 1200 Brussels, Belgium; 3 Louvain Centre for Toxicology and Applied Pharmacology (LTAP), Université Catholique de Louvain, 1200 Brussels, Belgium

**Keywords:** COVID-19, ARDS, Mechanical ventilation, Ketamine/esketamine, Prolonged infusion, Cholestasis, Cholangitis

## Abstract

**Background:**

To investigate the possible influence of prolonged ketamine (K) or esketamine (ESK) infusion on the profile of liver cholestatic biomarkers in patients with COVID-19 infection.

**Methods:**

A retrospective analysis was performed on 135 patients with COVID-19 related ARDS who received prolonged K or ESK infusion. They were compared to 15 COVID-19 ICU patients who did not receive K/ESK while being mechanically ventilated and 108 COVID-19 patients who did not receive mechanical ventilation nor K/ESK. The profile of the liver function tests was analysed in the groups.

**Results:**

Peak values of ALP, GGT and bilirubin were higher in the K/ESK group, but not for AST and ALT. Peak values of ALP were significantly higher among patients who underwent mechanical ventilation and who received K/ESK, compared with mechanically ventilated patients who did not receive K/ESK. There was a correlation between these peak values and the cumulative dose and duration of K/ESK therapy.

**Conclusions:**

Based on the observations of biliary anomalies in chronic ketamine abusers, prolonged exposure to ketamine sedation during mechanical ventilation may also be involved, in addition to viral infection causing secondary sclerosing cholangitis. The safety of prolonged ketamine sedation on the biliary tract requires further investigations.

**Supplementary Information:**

The online version contains supplementary material available at 10.1186/s12871-023-02006-2.

## Introduction

Optimal analgesia and sedation in patients with COVID-19 requiring prolonged mechanical ventilation in the Intensive Care Unit (ICU) remain challenging [1]. The quantity of sedation used in this population appears much higher than that usually prescribed in patients with adult respiratory syndrome (ARDS) from another origin [2]. Ketamine (K) is a N-methyl-D-aspartate (NMDA) receptor antagonist that can be used at sub-anesthetic doses for its analgesic and sedative dose-sparing effects in mechanically ventilated patients [3]. Esketamine (ESK) is the S( +) enantiomer of K. The use of K is usually limited by its psychomimetic effects, but short-term K infusion is usually not associated with liver or biliary injury, contrasting with the potential toxicity observed in long-term K abusers. On the other hand, COVID-19 itself has been associated with abnormal liver tests. There is an increasing number of reports of a progressive cholestatic injury in mechanically ventilated COVID-19 patients receiving K. The objective of this work was to compare the profile of liver cholestatic biomarkers in patients with COVID-19 receiving prolonged K or ESK infusion in the multimodal sedation regimen to the profile of COVID-19 patients, mechanically ventilated or not, who did never receive K/ESK. Patients with pre-existing liver disease were excluded from the analysis, but we describe shortly the case of a young patient with non-alcoholic steatohepatitis who presented a progressive cholestatic injury and required liver transplantation, as an illustration of the susceptibility of some subgroups of patients to this complication of SARS-CoV-2 infection.

## Material and methods

### Population

A retrospective analysis was performed on 258 patients with COVID-19 who were admitted to the ICU during the consecutive epidemic waves between March 1, 2020 and March 1, 2022. The study was approved by the Institutional Review Board (IRB) and a written informed consent was waived for a retrospective analysis based on the chart data. On the whole, 307 patients were admitted in the ICU over this period. However, 49 patients were excluded from the analysis for the following reasons: diagnosis of COVID-19 pneumonia not confirmed by RT-PCR (*n* = 2), incomplete chart data (*n* = 5), pre-existing liver disease (*n* = 19), acute liver failure related to shock (cardiogenic, septic, haemorrhagic) during ICU stay (*n* = 23).

### Pharmacological management and ventilation

Among the 258 remaining patients, 135 patients with COVID-19 related ARDS received during the period of mechanical ventilation a multimodal sedation regimen including propofol, sufentanil, midazolam, and K/ESK. Clonidine was given as adjunctive therapy to some patients. Cisatracurium was used as neuromuscular blocking agent at least during the first week of mechanical ventilation. K was infused at a rate ranging from 0.25 to 0.5 mg/kg/hour. When availability of K was reduced, ESK was used at the rate of 0.125 to 0.25 mg/kg/hour. Specific treatment for COVID-19 included hydroxychloroquine during the first period of ICU admission, and dexamethasone for the last periods. Other medications, including antibiotics, were administered according to patient’s condition. All patients received enteral nutrition.

Patients were mechanically ventilated targeting a tidal volume below 6 ml/kg ideal body weight, a plateau pressure < 30 cmH_2_O and a driving pressure < 15 cmH_2_O, under permissive hypercapnia with a lower pH limit of 7.25 in order to ensure an arterial haemoglobin saturation above 88%. Ventilation in prone position and administration of inhaled nitric oxide (NO) were also allowed.

### Data collection

Liver tests included serum aspartate transferase (AST), alanine transferase (ALT), alkaline phosphatase (ALP), gamma-glutamyl transferase (GGT), total bilirubin (TBIL). The laboratory reference upper limit of normal value (ULN) was: AST > 36; ALT > 35, ALP > 105, GGT > 40, TBIL > 1.2.

The distribution of liver function tests was reported as within the normal range and two times higher than the upper limit of normal (ULN) in three time points during ICU stay.

Ultrasound examination of the hepatobiliary tract was performed when indicated clinically.

### Statistical analysis

A comparison was done with 15 COVID-19 patients who never received K/ESK during mechanical ventilation and 108 COVID-19 patients who never received K/ESK as high-flow nasal oxygen therapy was sufficient to control hypoxemia. For the comparison, the patients who received K, ESK, or both, were considered as a single group, at the exception of the calculation of the cumulative dose for the patients who received both drugs.

Statistical analyses were performed using SPSS 21 (IBM SPSS Statistics for Windows, Version 21.0. Armonk, NY) and figures were created using Graphpad Prism 9 (GraphPad Software, LaJolla, CA). Values were expressed as median (first-third quartiles) for continuous values and counts (per percent of group) for qualitative variables. The data were subjected to Kolmogorov–Smirnov normality test and Bartlett’s test for homogeneity of variance. We compared clinical, demographic variables and relevant biochemical analysis between the three group of patients (IMV + /Ketamine + vs IMV + /Ketamine- vs IMV-/Ketamine-), using the chi-squared test (or Fisher’s exact test when appropriate) for categorical variables and Kruskall-Wallis test (with a Dunn’s post-test to correct for multiple comparisons) for quantitative data. Relevant biochemical values between K/ESK groups were compared using unpaired T test. All tests were two-sided, with significance level set at 0.05.

## Results

Baseline characteristics and outcomes of the whole population (*n* = 258) are shown in Table 1. Among the type of ketamine administered, 60.7% of the patients received exclusively K, 26.7% exclusively ESK and 12.6% both K and ESK. Cumulative dose was expressed only for the patients who received exclusively K or ESK, as no exact equivalence could be determined in case of combined therapy. The results of liver tests are presented in Table II.

**Table 1:** Baseline characteristics and outcome of the study population

Variables	COVID + /IMV/Ketamine + (*n* = 135)	COVID + /IMV + / Ketamine – (15)	COVID + /IMV-/ Ketamine – (108)
**Age, median (IQR), years**	64 (55–72)	68 (58–74)	61 (53–71)
**Male, *n* (%)**	94 (69.6)	13 (86.7)	80 (74.1)
**Alcohol abuse, *n* (%)**	4 (3)	0	5 (4.6)
**APACHE II, median (IQR)**	15 (12–18)	18 (16–20)^*^	13 (10–16)^*&^
**SOFA, median (IQR)**	6 (4–8)	6 (5–8)	4 (3–5)^*^
**Invasive mechanical ventilation, *n* (%)**	135 (100)	15 (100)	0^*&^
**Vasoactive drugs, *n* (%)**	102 (75.6)	14 (93.3)	2 (1.9)^*&^
**CRRT, *n* (%)**	34 (25.2)	2 (13.3)^*^	3 (2.8)^*&^
**Adjunctive treatments, *n* (%)**			
** Steroids**	87 (64.4)	15 (100)^*^	78 (72.2)
** Hydroxychloroquine**	26 (19.3)	7 (46.7)	19 (17.6)
** Azithromycine**	3 (2.2)	0	2 (1.9)
** Duration of sedation, median (IQR), days**	22 (11–35)	9 (3–18)^*^	0^*&^
**Type of Ketamine used,** ***n*** **(%)**			
** Ketamine**	82 (60.7)		
** Esketamine**	36 (26.7)		
** both**	17 (12.6)		
**ICU mortality,** ***n*** **(%)**	81 (60)	10 (66)	19 (17.6)^*&^

*CRRT * Continuous renal replacement therapy,  *IMV * Invasive Mechanical Ventilation, *IQR * Interquartile range, *SOFA * Sequential Organ Failure Assessment,  *APACHE II * Acute Physiology and Chronic Health Evaluation II, *LOS * Length of Stay

^*^indicates *p* < 0.05 in comparison with COVID + /IMV + /Ketamine + ; ^&^ indicates *p* < 0.05 in comparison with COVID + /IMV + /Ketamine-

**Fig. 1:**
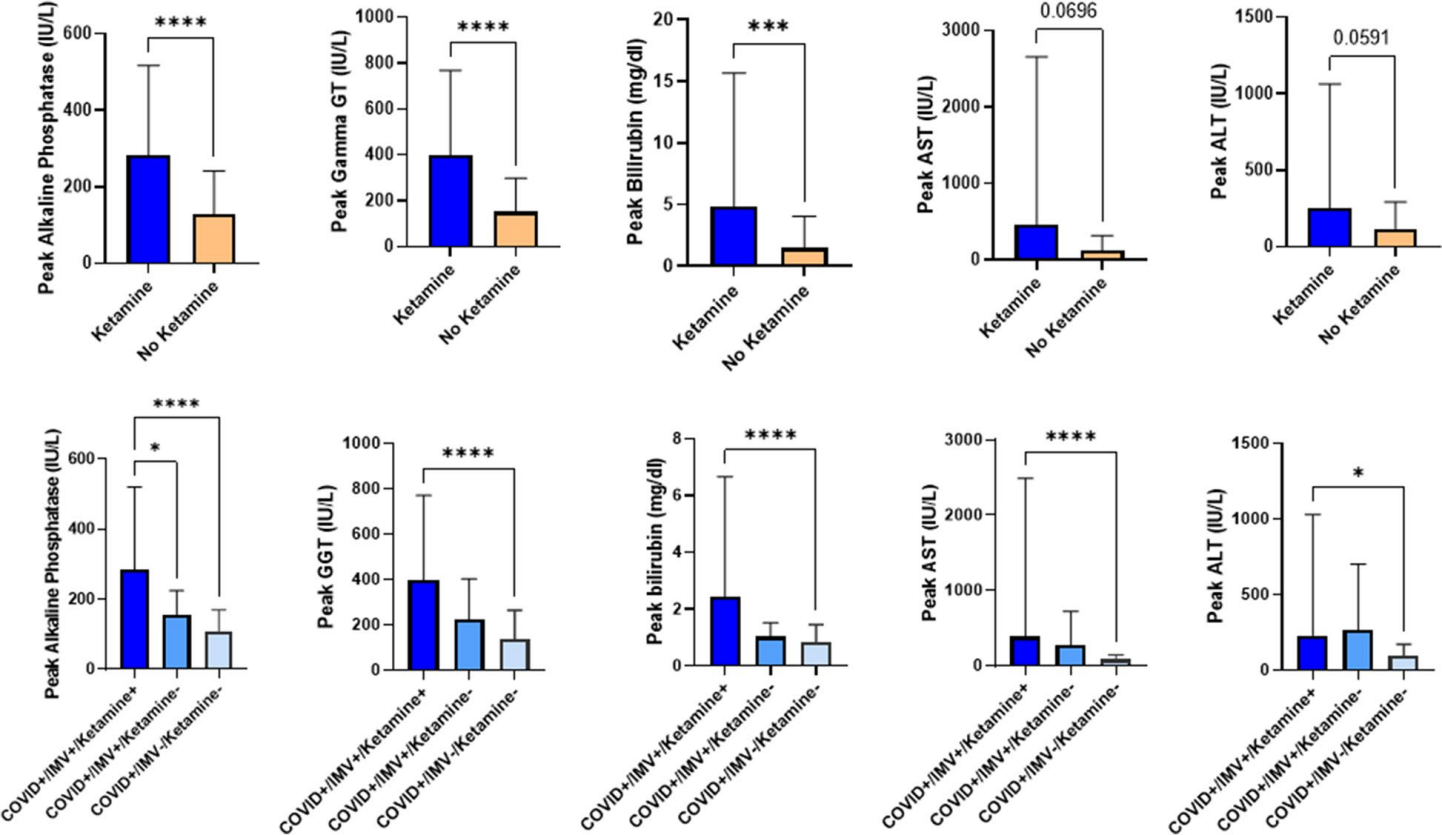
Comparison of peak ALP, GGT, bilirubin, AST and ALT in the ketamine/esketamine (E/ESK) + or – groups (upper part). Results expressed according to invasive mechanical ventilation (IMV) + or -, and K/ESK + or – (lower part).* indicates *p* < 0.05; ** indicates *p* < 0.005; *** indicates *p* < 0.0005; **** indicates *p* < 0.0001

**Fig. 2:**
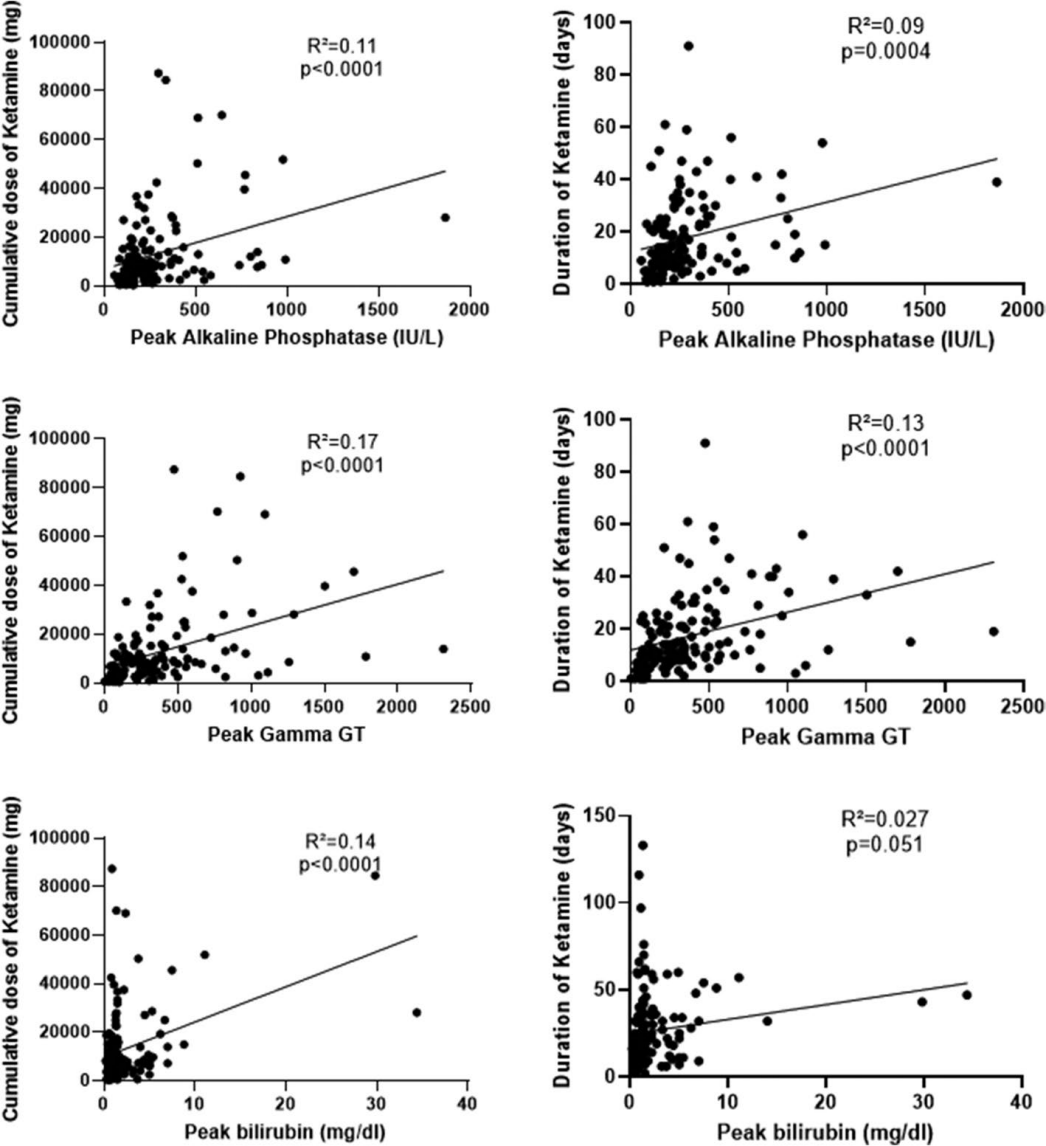
Peak of ALP, GGT and bilirubin according to the cumulative dose of K/ESK (left) and duration of K/ESK therapy (right). Cumulative dose was not calculated for the patients who received combined K/ESK therapy

The comparison between K/ESK versus no K/ESK group showed that the peak value for ALP, GGT and bilirubin was significantly higher in the K/ESK group (p value respectively < 0.0001, < 0.0001, = 0.0005) (Table 2, Fig. 1). No significant difference existed for peak AST (*p* = 0.069) and ALT (*p* = 0.059) values, despite a trend towards higher value in the K/ESK group. When looking at subgroups, patients with K/ESK and mechanical ventilation had a higher peak ALP value than the patients with IMV but without K/ESK (*p* = 0.018) and the patients who did not require neither mechanical ventilation nor K/ESK (*p* < 0.0001). For the other peak values (GGT, bilirubin, AST, ALT), the difference was only significant between the mechanically ventilated patients with K/ESK and the patients without mechanical ventilation and K/ESK. There was no significant difference regarding peak values of ALP, GGT, bilirubin, AST and ALT between patients who received K, ESK or both (Supplementary material). Besides, there was a moderate correlation between the cumulative dose of K/ESK over the ICU stay and the peak value for ALP (Pearson’s correlation coefficient R = 0.33, *p* < 0.0001)’s, GGT (R = 0.41, *p* < 0.0001) and bilirubin (R = 0.37, *p* < 0.0001) (Fig. 2). The same observation applied to the correlation between the duration of K/ESK therapy and peak values for ALP (R = 0.3, *p* = 0.0004) and GGT (R = 0.36, *p* < 0.0001) (Fig. 2). The median “time to peak value” of ALP (for the values at least two times higher than ULN, n = 85) was 14 days (IQR 7–23).

**Table 2:** Liver biomarkers in the study population

Variables	COVID + /IMV + / Ketamine + (*n* = 135)	COVID + /IMV + / Ketamine – (*n* = 15)	COVID + /IMV–/ Ketamine – (*n* = 108)
**Baseline ALP, median (IQR), IU/L**	69 (54–90)	66 (47–82)	60 (50–91)
**Number of cases with ALP > ULN at baseline (*n*** **=) (%)**	21 (16)	3 (20)	16 (15)
**Baseline GGT, median (IQR), IU/L**	54 (30–100)	56 (22–82)	54 (34–113)
**Number of cases with GGT > ULN at baseline (*n*** **=) (%)**	81 (60)	9 (60)	70 (65)
**Baseline bilirubin, median (IQR), mg/dl**	0.5 (0.4–0.7)	0.7 (0.5–0.8)	0.5 (0.4–0.7)
**Number of cases with bilirubin > ULN at baseline (*n*** **=) (%)**	8 (6)	1 (7)	1 (1)
**Baseline AST, median (IQR), IU/L**	48 (34–70)	37 (25–73)	39 (28–61)
**Number of cases with AST > ULN at baseline (*n*** **=) (%)**	93 (69)	8 (53)	63 (58)
**Baseline ALT, median (IQR), IU/L**	34 (19–50)	39 (17–63)	31 (21–52)
**Number of cases with ALT > ULN at baseline (*n*** **=) (%)**	65 (48)	8 (53)	44 (41)
**Peak ALP, median (IQR), IU/L**	218 (153–313)	150 (96–179)^*^	91 (54–145)^*^
**Number of cases with peak ALP > 2 × reference value, (*n*** **=) (%)**	71 (52.6)	2 (13)^*^	7 (6.5)^*^
**Peak GGT, median (IQR), IU/L**	305 (151–495)	185 (80–324)	88 (52–175)^*^
**Number of cases with peak GGT > 2 × reference value, (*n*** **=) (%)**	119 (88.2)	11 (73.3)	57 (52.7)^*^
**Peak bilirubin, median (IQR), mg/dl**	1.3 (0.8–2.2)	1.2 (0.6–1.3)	0.7 (0.5–0.9)^*^
**Number of cases with peak bilirubin > 2 × reference value, (*n* =) (%)**	29 (21.5)	0^*^	5 (4.6)^*^
**Peak AST, median (IQR), IU/L**	113 (68–168)	171 (65–258)	62 (39–104)^*^
**Number of cases with peak AST > 2 × reference value, (*n* =) (%)**	99 (73.3)	10 (66.7)	47 (43.5)^*&^
**Peak ALT, median (IQR), IU/L**	88 (56–166)	118 (55–274)	72 (42–112)^*^
**Number of cases with peak ALT > 2 × reference value, (*n*** **=) (%)**	86 (63.7)	11 (73.3)	55 (50.9)^*&^
**Number of cases with abnormal liver US, (*n *=) (%)**	19 (14.1)	1 (6.7)^*^	2 (1.9)^*^

*IMV * Invasive Mechanical Ventilation, *IQR * Interquartile range, *ALP * Alkaline phosphatase, *GGT * Gamma-Glutamyltransferase, *AST * Aspartate aminotransferase,  *ALT *Alanine aminotransferase, *ULN * Upper limit of normal value, *US * Ultrasound

^*^indicates *p* < 0.05 in comparison with COVID + /IMV + /Ketamine + ; ^&^ indicates *p* < 0.05 in comparison with COVID + /IMV + /Ketamine-

Among the patients who developed abnormal cholestatic biomarkers during ICU stay, 38 were investigated by abdomen ultrasonography. Examination was normal in 16 patients, abnormal in 22 patients, predominantly in the K/ESK group. The most frequent anomaly was gallbladder sludge (*n* = 14), followed by liver steatosis (*n* = 5), gallbladder hydrops (*n* = 5), and acute cholecystitis (*n* = 2). In the K/ESK group, two patients needed percutaneous drainage of the gallbladder and one patient required additional surgical drainage for biliary peritonitis.

**Fig. 3:**
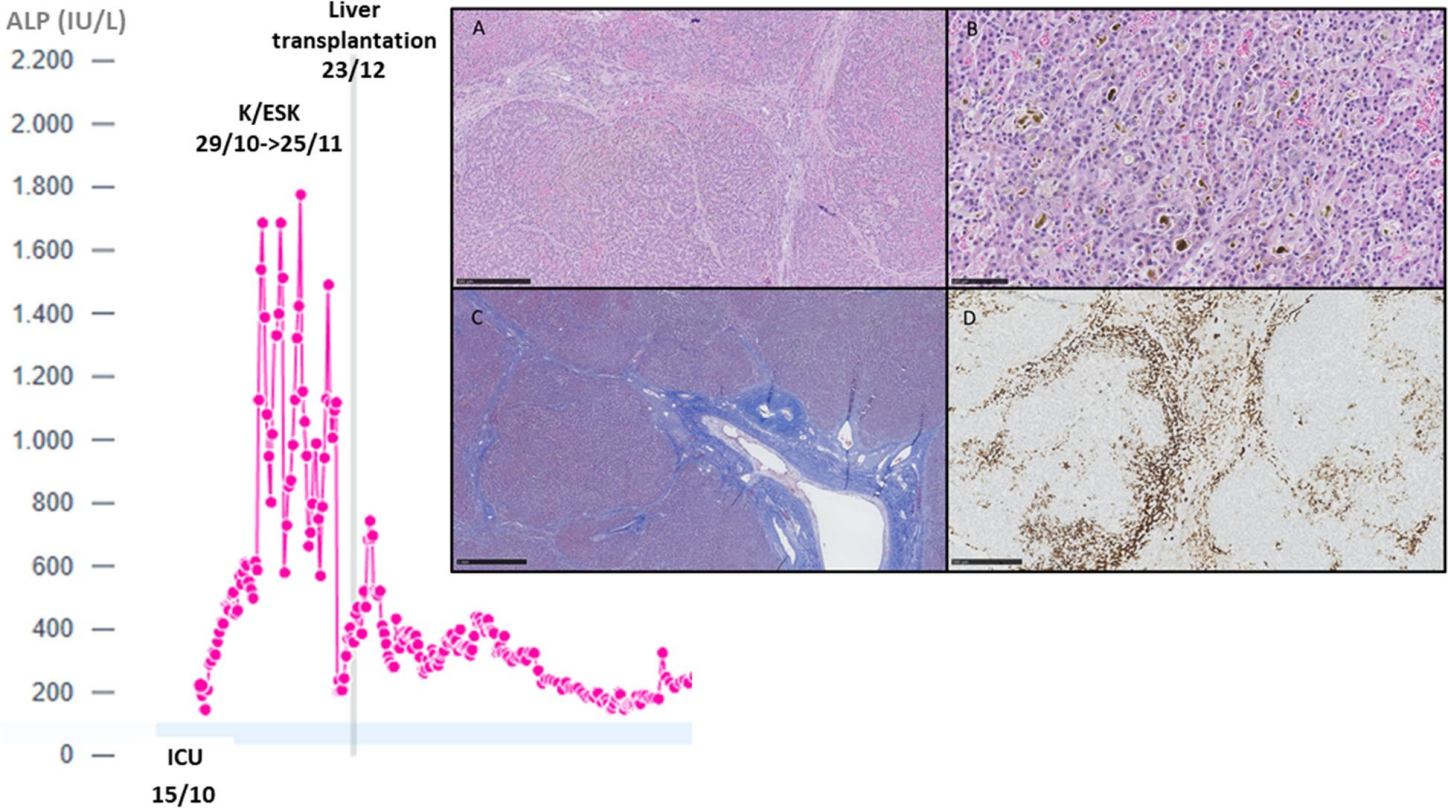
Evolution of plasma ALP in the 30-year-old cirrhotic woman who received both K and ESK over 28 days (left). Ultrastructural examination of the explanted liver (right). The histological sections on the explanted liver showed a nodular architecture associated with sinusoidal congestion (A, H&E 5X), bilirubinostasis with biliary thrombi (B, H&E 20X) and septal fibrosis (C, Masson Trichrome 5X). Not significant inflammation was observed in portal tracts. Immunohistochemistry for cytokeratin 7 showed a severe cholestasis and canalicular proliferation (CK7, 5X)

Among the patients who were excluded from the analysis for pre-existing liver disease, a 30-year-old woman had a Child–Pugh B liver cirrhosis as an unusual complication of Turner syndrome (non-alcoholic steatohepatitis (NASH)). When mechanically ventilated for COVID-19 related ARDS, she received a cumulative dose of 2040 mg of K and 2370 mg of ESK over a period of 28 days. We noted a marked increase in plasma ALP levels (Fig. 3, left). Due to the progression of liver failure, a liver transplantation was required 28 days after the cessation of ESK. Ultrastructural examination of the explanted liver was consistent with a cholestatic injury (Fig. 3, right). The responsibility of K/ESK remained speculative as the patient also experienced infectious complications, but without shock.

Otherwise, in this group of COVID-19 patients with K/ESK, no patient required a liver transplantation and a progressive decrease of ALP levels was noted in most of the patients who survived.

## Discussion

Ketamine is usually considered as a safe anaesthetic agent in the operating room. By contrast, studies having investigated the safety of ketamine in critically ill patients have classically considered infusion of ketamine for periods below 72 h [3–5]. With prolonged infusion in doses above 1 mg/kg/h, there is a concern about the possible development of hepatobiliary injury that is usually nor observed after short exposure periods. Before the era of COVID-19, such description was limited to burned patients having received prolonged ketamine infusion in the ICU or to patients having abused ketamine over periods exceeding sometimes 10 years. In a large cohort of 293 burned patients who required mechanical ventilation, a higher incidence of cholestasis or proven secondary sclerosing cholangitis (SSC) was observed in the subgroup that received a ketamine-based sedation [6]. Ketamine has also been used off-label for several chronic outpatient diseases, such as treatment-resistant depression and chronic pain syndromes. In a retrospective case series analysis combined to medical literature data, Cotter et al*.* reported an association between repeated or continuous administration of ketamine and adverse hepatobiliary events ranging from reversible asymptomatic elevation in both serum liver aminotransferases and ALP to the development of permanent structural changes including cirrhosis and pericholeductal fibrosis [7].

Long-term exposure in chronic ketamine abusers has been associated with cholestasis, biliary dilatation, biliary sepsis and decompensated cirrhosis. In a survey of 257 consecutive chronic abusers of ketamine with urinary tract dysfunction, the prevalence of biliary tract anomalies on MRCP was 61.9% [8]. A reversal of liver cholestatic biomarkers may be observed after ketamine withdrawal [9].

The mechanism of ketamine liver toxicity is thought to be either by direct action on the biliary smooth muscle and by central action [10, 11]. The direct effect on the smooth muscle cells could be explained by the activation of NMDA receptors located on smooth muscle cells which can cause their contraction, the antagonisation obtained by ketamine on those receptors could cause their relaxation and dilatation of the biliary system [12]. Ketamine can also induce a contraction of the sphincter of Oddi, impairing the outflow of bile and could therefore further exacerbate the bile accumulation [13]. The central effect can be explained by blockade of the NMDA receptors in the dorsal motor nucleus of the vagus nerve, possibly inducing gallbladder dyskinesia [13]. These different factors can lead to bile stasis and promote the precipitation of norketamine in the bile which can then lead to biliary obstruction, cholangitis and finally secondary biliary cirrhosis [14].

During the recent COVID-19 pandemic, ventilated patients suffering from ARDS usually required high sedative and analgesic doses for prolonged periods of time and, after the shortage of common anaesthetic agents, long-term, high dose infusion of ketamine was frequently adopted as adjunctive agent. However, many ICU physicians had the clinical impression of a progressive cholestatic liver injury in mechanically ventilated COVID-19 patients receiving ketamine. Among the varying degrees of cholangiopathies, the pattern of SSC was more particularly investigated. It remains to be determined if cholangiopathy was related to the infection by SARS-CoV-2, to the consequences of a hypoxic/ischemic injury (not specifically induced by SARS-CoV-2 infection) or to a drug-induced injury. Previous publications have identified that increased liver tests (mainly AST and ALT) may be found in 14–78% of individuals affected by COVID-19 [15–18]. The pattern of liver injury seems predominantly hepatocellular rather than cholestatic, as indicated by the milder elevation of total bilirubin and alkaline phosphatase in most of the publications [19]. In addition, abnormal liver function tests were more frequently observed in the patients who required ICU admission. Cholestatic liver function tests were incompletely reported in 510 patients [20]. Mean levels of ALP were 71 IU/L across three studies, and mean levels of GGT were 40.6 IU/L across four studies [17, 21, 22]. Mean levels of ALP were 71 IU/L across three studies, and mean levels of GGT were 40.6 IU/L across four studies [17, 21, 22]. At least, two types of cholangiopathies have been associated with SARS-CoV-2 [23]. The first form has probably an autoimmune origin resembling primary biliary cholangitis (PBC), while the second has the characteristics of a secondary sclerosing cholangitis (SSC) [14, 24–30]. The diagnosis can be made by magnetic resonance cholangiopancreatography (MRCP) showing dilatations and strictures of intrahepatic bile ducts, with alterations of the common bile duct. This last condition was mainly observed in the most severe ICU patients with ARDS, renal and cardiocirculatory support. Also patients with non-alcoholic fatty liver disease (NAFLD)/NASH and metabolic risk factors seem at particular risk for developing cholestatic liver failure and/or SSC after COVID-19 [31]. The progressive increase of ALP and GGT is usually contrasting with the modest increase in AST and ALT. Liver transplantation was required in some patients [32].

The potential role of ketamine is supported by several observations [33–35]. During the first pandemic wave in Europe (March–April, 2020), five COVID-19 patients presented a dose-dependent, progressive cholangiopathy after a mean duration of 16 (6–26) days of intravenous ketamine administration, with a mean cumulative dose of 9.5 (3.8–95.9) grams. Histological features of cholangitis were documented by liver biopsy in 4/5 patients [14]. In another study, a comparison of 34 intubated patients with COVID-19 and 34 intubated patients with influenza revealed that four developed SSC in the first group and none in the second group; ketamine was used for sedation in the COVID-19 group, and not in the second group [34]. The largest series was published by Wendel-Garcia et al. who compared 170 COVID-19 ventilated patients who received ketamine infusion to 73 patients who did not [35]. Patients having received ketamine infusion had a multivariable adjusted competing risk hazard of developing cholestatic liver injury during their ICU stay of 3.2. Our observations also found a correlation between the cumulative dose of K/ESK over the ICU stay and the peak value for ALP, GGT and bilirubin. We also confirmed that a delay of about 14 days is usually observed before the peak of ALP value [35].

The limitations of this retrospective analysis have to be acknowledged. First of all, the severity of the non-ventilated COVID-19 patients who never received ketamine was lower as expressed by the SOFA and APACHE II scores on admission. These patients had also a lower rate of complications (need for CRRT, vasoactive drugs) and a lower mortality rate. The severity of the COVID-19 ARDS patients who were mechanically ventilated without ketamine use was however similar as that of the ketamine group, but the number of observations was limited. Numerous drugs can induce a cholestatic reaction, but the most commonly used medications (other sedatives, antithrombotic agents,..) were equally balanced among the mechanically ventilated patients, with or without ketamine. Other causes may have contributed to biliary tract injury, including systemic inflammatory response syndrome, prolonged hypoxia or refractory shock, as SSC has also been described in critically ill patients needing mechanical ventilation for another aetiology than COVID-19 [36]. We excluded from our analysis the patients who presented an acute liver injury in relationship with shock from any aetiology (cardiogenic, septic,…). The number of patients who had extensive serological investigations (virus, auto-immunity, …) was also limited. However, the role of ketamine is still supported by the relationship with the time to peak for ALP (without significant changes in AST and ALT), and with the cumulative dose and total duration of ketamine therapy.

## Supplementary Information

**Additional file 1.**

## Data Availability

The datasets used and/or analysed during the current study are available from the corresponding author on reasonable request.
