# Supplementary figures and images for: Profile of liver cholestatic biomarkers following prolonged ketamine administration in patients with COVID-19
Source: BMC Anesthesiol. 2023 Feb 7;23:44. doi: 10.1186/s12871-023-02006-2 (PMC9902832; doi:10.1186/s12871-023-02006-2)

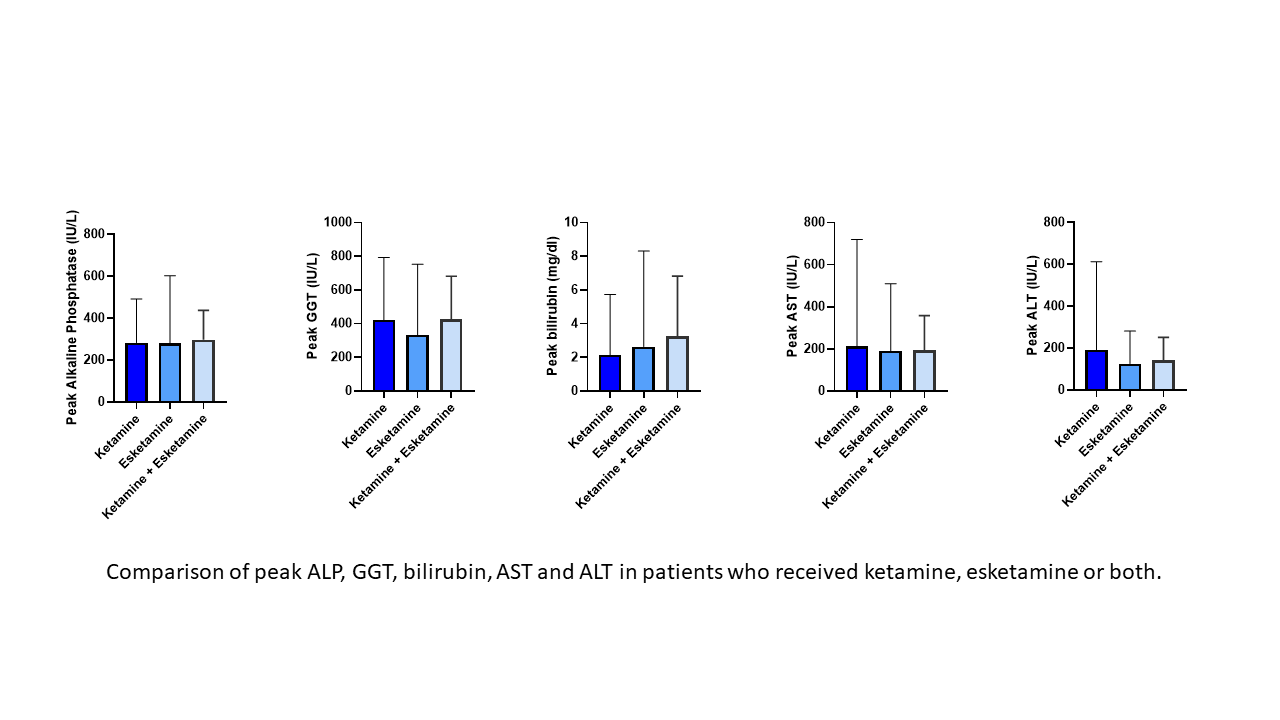

Supplement: Supplementary file 1 — Additional file 1. [file 12871_2023_2006_MOESM1_ESM.tif]
